# High-Protein Diets during either Resistance or Concurrent Training Have No Detrimental Effect on Bone Parameters in Resistance-Trained Males

**DOI:** 10.3390/nu16020325

**Published:** 2024-01-22

**Authors:** Reza Bagheri, Zohreh Karimi, Zeynabalsadat Mousavi, Mahdi Ziaee Bashirzad, Donny M. Camera, Ramin Sadeghi, Vahid Reza Dabbagh, Mehdi Kargarfard, Frederic Dutheil

**Affiliations:** 1 Department of Exercise Physiology, Faculty of Sport Sciences, University of Isfahan, Isfahan 8174673441, Iran; will.fivb@yahoo.com; 2 Department of Physical Education and Sport Sciences, Science and Research Branch, Islamic Azad University, Tehran 1477893855, Iran; zohreh.karimi1@gmail.com; 3 Nutrition and Food Service, Imam Khomeini Hospital Complex, Tehran University of Medical Sciences, Tehran 1416634793, Iran; zeynabmousavi0@gmail.com; 4 Department of Sport Science, Islamic Azad University, Bojnourd Branch, Bojnourd 9417697796, Iran; mziaee1981@gmail.com; 5 Department of Health and Biostatistics, Swinburne University, Melbourne, VIC 3122, Australia; dcamera@swin.edu.au; 6 Nuclear Medicine Research Center, Mashhad University of Medical Sciences, Mashhad 9177949025, Iran; sadeghir@mums.ac.ir (R.S.); dabbaghvr@mums.ac.ir (V.R.D.); 7 Université Clermont Auvergne, CNRS, LaPSCo, Physiological and Psychosocial Stress, CHU Clermont-Ferrand, University Hospital of Clermont-Ferrand, Preventive and Occupational Medicine, Witty Fit, F-63000 Clermont-Ferrand, France; fred_dutheil@yahoo.fr

**Keywords:** concurrent exercise, resistance training, protein availability, bone health, nutrition

## Abstract

Background: The effects of combining resistance training (RT) and concurrent training (CT; resistance + endurance training) with varied protein doses on bone measures remain poorly understood. Hence, we conducted a comparison of the impacts of two high-protein diets (1.6 or 3.2 g kg^−1^ d^−1^) over 16 weeks in resistance-trained males, either with CT or RT alone. Methods: A total of forty-eight males, all of whom were resistance-trained, had the following demographics: 26.6 ± 6 years, body mass index: 25.6 ± 2.9 kg m^−2^ administered either 3.2 g kg^−1^ d^−1^ protein (CT2; *n* = 12; RT2; *n* = 12) or 1.6 g kg^−1^ d^−1^ protein (CT1; *n* = 12; RT1; *n* = 12) during 16 weeks (four sessions·w^−1^). Bone parameters were assessed pre- and post-intervention. Results: There was no significant interaction between the intervention group and time for the legs, arms, ribs, or pelvis area BMC and BMD (*p* > 0.05). For the BMD of the pelvis and the BMC of the right ribs, however, there were significant time effects noted (*p* < 0.05). Furthermore, there was a significant interaction between the intervention group and time in the lumbar and thoracic spines, with a particular time effect noted for the thoracic spine region (*p* < 0.05). The regional differences in skeletal responses to the intervention are highlighted by these data. Conclusion: Our findings show that the intake of two high-protein diets combined with RT and CT during 16 weeks had no adverse effects on bone tissue parameters. While these findings indicate that protein intake between 2 and 3 times the current RDI does not promote bone demineralization when consumed in conjunction with exercise, future studies investigating the long-term effects of chronic high protein intake on bone tissue health are warranted.

## 1. Introduction

Resistance training (RT) has been proven to be the optimal training modality for enhancing anabolic-related changes, such as muscular strength, power, and endurance, among trained adults [1]. In contrast, it has been shown that endurance training (ET) may facilitate enhancements in VO_2max_ with augmented cardiovascular health and function, as well as increased skeletal muscle oxidative capacity [2]. Given that the training adaptations of ET and RT differ substantially and can be influenced by factors such as the type, intensity, and volume of exercise, it seems logical to incorporate both modalities into a unified training program to optimize anabolic, metabolic, and oxidative adaptation responses simultaneously. Concurrent training (CT) is often defined as the integration of RT and ET within a single training regimen. Prior research has shown that the use of CT has the potential to enhance several aspects of physical performance, including muscular strength, anaerobic power, aerobic capacity, and maximum velocity contraction responses [3,4,5].

Developing and maintaining skeletal muscle mass and function is also pivotal to bone strength and mineral density [6]. Bone mineral density (BMD) is a measure of the amount of bone mineral content (BMC) per unit of area. It is used to assess the strength and quality of bone tissue, serving as an indicator of its ability to undergo structural remodeling [7]. There is strong evidence indicating that engaging in physical activity throughout early adulthood has a beneficial impact on the accumulation of bone and the enhancement of maximum bone mass during the third decade of life [8]. Mechanical loading is generally believed to exert a crucial effect on bone [9,10]. Previous studies have shown that skeletal muscle tissue seems to influence bone health [11,12] and factors produced by muscle contractions [12,13]. Gravity-derived loads (impact training) and muscle-derived loads such as RT have both produced positive effects on bone in young women [14]. Therefore, modifying the mode, duration, and intensity of physical activity may serve as a viable strategy to enhance the bone health of athletes [15]. However, which source of loading supplies the most effective stimulus, gravitational impact loads or muscle forces, is equivocal [16]. Marques et al. (2011) demonstrated that eight months of RT was more effective than ET and yielded favorable changes in BMD and muscle strength [17]. ET is also used to treat osteoporosis because it has a low risk for fractures due to its low intensity coupled with the metabolic stimulus necessary for bone synthesis. Prior research has emphasized that RT is among the most efficacious non-pharmacological approaches to enhance (BMD) [18,19,20]. RT has been shown to promote bone formation and improve BMD and bone structure in children and adolescents [21,22,23]. The most significant skeletal advantages of RT have been achieved by gradually increasing the resistance over time, using a high magnitude of mechanical load (approximately 80% to 85% of the 1-repetition maximum [RM]), performing exercises at least twice a week, and targeting large muscles that cross the hip and spine [24]. It has been suggested that RT, or strength training, which includes high muscle and joint compressive forces, may have a more noteworthy positive influence on bone mass than other sorts of activities (e.g., running, swimming) [25]. Collectively, RT on its own or when combined with other therapies may be optimal for safeguarding against bone loss or perhaps enhancing BMD in both the lumbar spine and femoral neck [24].

There has been a notable emphasis on the impact of dietary protein on the fully developed skeletal system. This attention has been driven, at least in part, by a growing interest in nonpharmacological methods for preserving skeletal health into adulthood and later stages of life [26]. BMD, a primary determinant of bone strength, seems to exhibit a positive correlation with the intake of protein [27]. However, athletes who adhere to a high-protein diet may face the possible long-term consequences of promoting demineralization of the bone, which might have detrimental effects on bone health [15]. Multiple meta-analyses have sought to resolve the ongoing debate over the effectiveness of dietary protein intake on adult bone health. Darling et al. (2009) found no effect of higher protein intake on fracture outcomes [28], while the more recent study by Wu et al. (2015) found slight beneficial associations between high versus low intake on hip fracture risk [29]. Shams-White et al. (2017) reported a beneficial relationship between high (1.4 g kg^−1^ d^−1^) versus low (0.8 g kg^−1^ d^−1^) protein intake and BMD and BMC for nearly all bone sites; however, statistical significance was present only at the lumbar spine [26]. Contrary to long-held beliefs, these systematic reviews indicate that consuming increased amounts of dietary protein does not have any negative effects on bone health [26,28,29]. Considering the effects of a combination of chronic exercise training combined with a high-protein diet on the parameters of bone health are not entirely known, we compared the effects of two high-protein diets (1.6 or 3.2 g kg^−1^ d^−1^) during 16 weeks of either CT or RT alone on bone parameters in resistance-trained males. We hypothesized whether RT or CT combined with high-protein diets would affect bone parameters in resistance-trained males. 

## 2. Methods 

### 2.1. Participants

The current investigation enlisted a sample of 48 male participants who were young, healthy, and engaged in RT. The same participant cohort was used in a previous publication that investigated the effects of different high protein intakes on body composition, muscular strength and performance, and markers of liver and kidney [30]. These individuals were between the ages of 18 and 36 and were recruited via the use of advertising on various social media platforms. This research and testing protocols were communicated to interested participants via either telephonic or in-person sessions held at nearby fitness facilities. Participants were instructed to complete a health and fitness history questionnaire, providing information about their previous training background, specifically engaging in three sessions per week for a minimum of one year of RT experience (with three to four sessions per week). Additionally, participants were required to report sleeping for a duration of seven to eight hours within a 24 h day, abstaining from the use of steroids or any illegal substances known to enhance muscle size for the past year, consuming less than ~1.6 g kg^−1^ d^−1^ of protein, and being free from any musculoskeletal disorders. Participants who met the aforementioned criteria provided both written and verbal consent to participate in this study. Furthermore, as part of the permission process, participants were provided with a medical history questionnaire, and they were requested to revisit the research site to complete this study procedures. The procedure underwent a thorough evaluation by the Institutional Human Subject Committee and the Ethics Committee of the University of Isfahan (IR.UI.REC.1400.098) and was conducted in strict adherence to the principles outlined in the Declaration of Helsinki. The present research has been duly filed with the Iranian Registry of Clinical Trials with the registration number IRCT20191204045612N2.

### 2.2. Study Design

As previously published [30], following the collection of baseline measures, participants underwent a familiarization process with this research tests and procedures. Subsequently, they were randomized to one of four groups using the use of an online resource, namely www.randomizer.org (accessed on 30 January 2022): CT + 1.6 g kg^−1^ d^−1^ of protein (CT1; *n* = 12), CT + 3.2 g kg^−1^ d^−1^ of protein (CT2; *n* = 12), RT + 1.6 g kg^−1^ d^−1^ of protein (RT1; *n* = 12), or RT + 3.2 g kg^−1^ d^−1^ of protein (RT2; *n* = 12). The initial planned duration of this research was six months; however, in response to the global outbreak of the Coronavirus Disease 2019 (COVID-19) pandemic, we made a voluntary decision to conclude this study after 16 weeks. Consequently, data were gathered at the first assessment and the 18th week (after 16 weeks of the intervention) at the same time of day, with a time difference of −1 h. The participants had two initial testing sessions: during the first session, questionnaires were filled out, while during the second session, measurements of bone parameters were conducted. Following the completion of these assessments, study participants engaged in an initial consultation with the researcher’s dietician. This meeting served as an opportunity to discuss their individual dietary preferences as well as establish specific protein and calorie consumption goals in preparation for the commencement of their respective training programs. All operations specified were executed in a strictly sequential manner for all time measurements. 

### 2.3. Anthropometry and Bone Parameters

In this study, participants were instructed to assume a supine position on the dual-energy X-ray absorptiometry (DXA) examination table, wearing shorts, for a duration of roughly 7 min. During this time, a low dosage of radiation was used to conduct a comprehensive scan of their complete body. The DXA scans were performed by a single technician using the Hologic APEX software, version 4.5.3.2, which includes the image comparison mode for serial evaluation to measure area, BMC, and BMD for the left arm, right arm, left ribs, right ribs, T spine, L spine, pelvis, left leg, and right leg. The scans were completed following the manufacturer’s requirements for calibration and testing protocols, as outlined in previously published research [31]. 

### 2.4. Resistance Training

The participants included in the two RT groups engaged in a structured exercise regimen consisting of four sessions per week. These sessions were conducted on certain days, namely Saturday, Monday, Wednesday, and Thursday. The training program followed a linear periodization approach and included two sessions targeting the upper body and two sessions targeting the lower body each week. Additionally, all sessions were monitored to ensure proper execution and adherence to the prescribed training program. Before each resistance training (RT) session, the participants engaged in a warm-up routine consisting of both general and specific activities. The general warm-up lasted for 10 min and involved either slow running on a treadmill at a speed of 3–5 km or using an elliptical machine at a level of 5–10. Following the general warm-up, participants performed a specific warm-up for 5 min. This specific warm-up included exercises such as medicine ball twists (1 set of 10 repetitions), medicine ball wood chops (1 set of 10 repetitions), straddled toe touches (2 sets of 5 repetitions), dynamic quadriceps stretches (1 set of 5 repetitions), and medicine ball squats (1 set of 5–8 repetitions).

Subsequently, the participants engaged in an upper-body RT regimen comprising seven exercises (chest press, lateral pulldown, standing barbell shoulder press, standing shoulder shrugs, bicep curl, triceps press down, and abdominal crunch) executed twice per week. The lower-body RT program, comprised of six exercises (seated leg curl, 45-degree leg press, back squats, barbell hip thrusts, back extension, and calf raises), was also performed twice per week. For weeks 1–4, participants completed three sets of twelve repetitions at 75% of their 1-RM; for weeks 5–8, three sets of ten repetitions at 80% of their 1-RM; for weeks 9–12, four sets of eight repetitions at 85% of their 1-RM; and for weeks 13–16, four sets of six repetitions at 90% of their 1-RM. The duration of rest intervals between sets and exercises did not exceed two minutes [32]. The periodized RT program was based on our previous work [32] and following recommendations by the National Strength and Conditioning Association [33]. Verbal encouragement and comments were provided to the participants both during and after each set. The training data for each participant were recorded, ensuring that the training intensity was optimized throughout each session and that participants effectively adopted progressive overload in a personalized manner. In addition, study personnel supervised all training throughout this study. 

### 2.5. Concurrent Training 

The participants in both CT groups engaged in a total of four sessions each week, namely on Saturday, Monday, Wednesday, and Thursday. Each session consisted of RT carried out at the start, followed by ET, as per the prescribed exercise order sequence [34] to minimize possible interferences in muscle anabolism. Before each CT session, the participants engaged in a warm-up routine consisting of both general and specific activities. The general warm-up involved 5 min of slow running on a treadmill or using an elliptical machine at a speed of 3–5 km. The specific warm-up activities lasted for 5 min and included exercises such as medicine ball twists (10 repetitions), medicine ball wood chops (10 repetitions), straddled toe touches (2 sets of 5 repetitions), dynamic quadriceps stretches (1 set of 5 repetitions), and medicine ball squats (1 set of 5–8 repetitions). The participants then engaged in the same RT program as previously stated. The participants engaged in endurance cycle training on ergometers immediately after completing RT. The training consisted of a combination of hill simulation rides with different intensities (ranging from 25 to 110 MAP), moderate-intensity continuous training at 50% MAP, moderate-intensity interval training (MICT) at 70% MAP, and high-intensity interval training (HIIT) at 100% MAP. At 40% MAP, moderate-intensity intervals were separated by a 60 s recovery period in order to determine whether the work-to-rest ratio was 2.5:1 or 5:1. Work-to-rest ratios of 1:5, 1:2, or 1:1 were determined by separating high-intensity intervals with 20 to 60 s recovery periods conducted at 40% MAP. Every cycling session commenced with a 3–5 min warm-up period at or below 50 W. In order to implement progressive overload, the number of intervals and relative intensity of the burden were altered continuously. 

### 2.6. Training Volume

The RT volume was determined using the specified formula for each session and reported on a weekly basis [35].

1. RT volume = [repetitions (n) × sets (n) × load or selected weight (kg)].
2. The volume of ET was determined using the following formula: Total ET volume: [work + rest].
3. Work: [Intensity × maximum aerobic power (MAP) × (set × duration [as noted in the training protocol] × 0.06)].
4. Rest: [Intensity × MAP × (set × duration [as noted in the training protocol] × 0.06)].
5. Intensity: percent of MAP; Set: number of repetitions of each session; Duration: spent time (minutes); 0.06: Convert watts to kilojoules

### 2.7. Diet

This study participants were instructed to record their food intake for a total of six consecutive 24 h periods. These periods included four weekdays that were not consecutive and two non-consecutive weekend days. The purpose of this data collection was to assess the participants’ typical protein consumption patterns. In order to facilitate the attainment of their desired protein intake (i.e., 1.6 or 3.2 g kg^−1^ d^−1^), participants consumed a 40 g of isolated whey protein (Wisser Nutrition, Isfahan, Iran) beverage upon cessation of every training session that comprised the following nutrition profile per scoop (28 g): calories, 110; total fat, 0.5 g; saturated and trans-fat; sugars and dietary fiber, 0 g; sodium, 50 mg; potassium, 112 mg; total carbohydrate, 2 g; protein, 24 g. The remaining amounts of protein were received from dietary sources, and the habitual consumption of protein remained consistent across all groups during the intervention. 

The decision to include the protein group with a daily intake of 1.6 g kg^−1^ d^−1^ was justified by the findings of Morton et al. (2018), who suggested that this specific quantity of protein intake would optimize improvements in fat-free mass (FFM) after RT [36]. Since there is presently no research examining the impact of protein availability above 2–2.2 g kg^−1^ d^−1^ on training adaptation responses with CT, our objective was to establish a distinct disparity in protein intake across the groups. Therefore, we decided to increase the initial dose of 1.6 g kg^−1^ d^−1^ to 3.2 g kg^−1^ d^−1^ for the high protein group being compared. We also made sure that this higher amount could be safely tolerated. Antonio and his colleagues have previously shown that this quantity (~2.51–3.32 g kg^−1^ d^−1^) does not have any detrimental impact on indicators of liver and renal function [37]. 

The participants engaged in regular consultations with a certified dietitian every two weeks. During these consultations, they received instructions on how to meet their protein and energy requirements. Specifically, they were advised to distribute their protein intake throughout the day across 4–7 meals, with each meal containing 20–40 g of protein. This approach aims to optimize muscle protein synthesis (MPS) [38,39]. This research included monitoring the macronutrient composition, with particular emphasis on total energy intake (TEI) and protein intake. It has been recommended that individuals maintain their carbohydrate and fat consumption within the Acceptable Macronutrient Distribution Range, which suggests a range of 45–65% of total energy intake for carbohydrates and 20–35% of total energy intake for fats. The participants were instructed to maintain a state of positive energy balance to mitigate any possible disruptions to anabolic adaptations caused by energetic stress [40,41]. Participants in this research maintained daily food records using mobile phone apps. Those with iPhones used the Easy Diet Diary software, version 6.0.28, developed by Xyris Software Pty Ltd. (Australia), while those with Android-based devices used the My Fitness Pal app, version 24.2.0, developed by MyFitnessPal Inc. (USA). The dietary intake data were analyzed using Diet Analysis Plus, version 10, from Cengage. This was carried out to guarantee consistency in the food database utilized for all studies.

### 2.8. Statistical Analysis

The sample size was determined using PASS.15 software, which employed an F test, repeated measures, and within-between interaction ANOVA. The analysis indicated that 40 participants were required to detect a medium effect (Cohen’s f = 0.25) with a significance level of α = 0.05 and 80% power for detecting changes in bone parameters following an exercise training intervention [42,43]. Prior to conducting statistical analysis, the normality of the distribution of all variables was assessed using the Shapiro–Wilk test. No missing values were observed at any time point. The mean (SD) was used to reflect the baseline characteristics between groups at PRE. The effects of training and dietary interventions on dependent variables were examined using a two × four analysis of variance (ANOVA) with repeated measurements. This analysis used the factors of time (pre-test vs. post-test) and group (CT1 vs. CT2 vs. RT1 vs. RT2) to assess the variations between the treatments over time. When the group-by-time interaction was significant, we used Bonferroni post hoc analysis to determine between-group differences. All analyses and figure production were performed using GraphPad Prism (version 8.4.3). 

## 3. Results

### 3.1. Participant Characteristics

A total of 112 individuals underwent assessments to determine their eligibility. Twenty-eight of them failed to satisfy the established criteria for inclusion, while 36 individuals expressed a lack of interest in participating after the first interview. One participant from each group withdrew from this research, citing reasons such as scheduling constraints, lack of interest, COVID-19, or musculoskeletal injury. Lastly, 44 participants remained for the final analysis. There were no statistically significant differences seen between the groups in terms of baseline characteristics (Table 1).

### 3.2. Bone Parameters

#### 3.2.1. Upper Body

**Figure 1:**
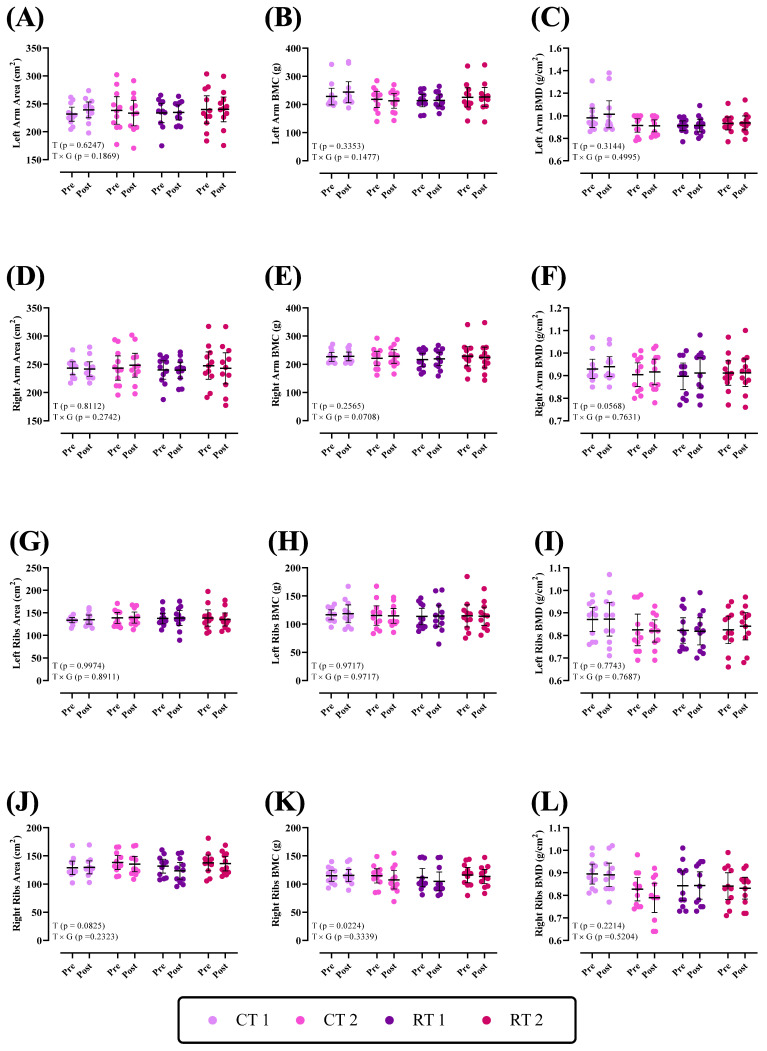
Effects of resistance or concurrent training in combination with high-protein diets on upper-body bone parameters. *n* = 11 per group; error bars represent 95% confidence interval (CI). CT1, concurrent training + 1.6 g kg^−1^ d^−1^; CT2, concurrent training + 3.2 g kg^−1^ d^−1^; RT1, resistance training + 1.6 g kg^−1^ d^−1^; RT2, resistance training + 3.2 g kg^−1^ d^−1^; T, time effect; T × G, time × group interaction; BMC, bone mineral content; BMD, bone mineral density.

Changes in bone parameters of the upper body throughout the intervention are shown in Figure 1. There was no group × time interaction (*p* > 0.05) for the left and right arm and ribs for area, BMC, and BMD (*p* values are shown in each figure). There was a significant time effect for right rib BMC (*p* = 0.0224). 

#### 3.2.2. Thoracic Spine and Lumbar

**Figure 2:**
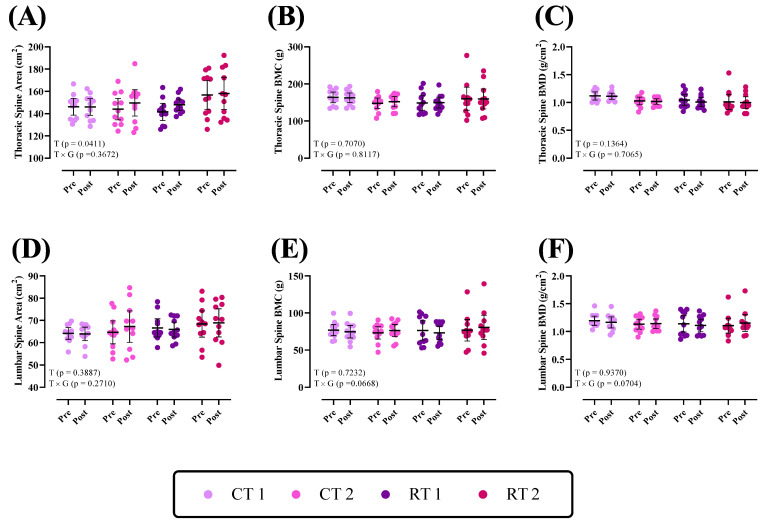
Effects of resistance or concurrent training in combination with high protein diets on Thoracic spine and Lumbar bone parameters. *n* = 11 per group; error bars represent 95% confidence interval (CI). CT1, concurrent training + 1.6 g kg^−1^ d^−1^; CT2, concurrent training + 3.2 g kg^−1^ d^−1^; RT1, resistance training + 1.6 g kg^−1^ d^−1^; RT2, resistance training + 3.2 g kg^−1^ d^−1^; T, time effect; T × G, time × group interaction; BMC, bone mineral content; BMD, bone mineral density.

Changes in bone parameters of the lumbar and thoracic spine throughout the intervention are shown in Figure 2. There was a group × time interaction (*p* > 0.05) for the lumbar and thoracic spine areas, BMC, and BMD (*p* values shown in each figure). However, there was a significant time effect for the thoracic spine area (*p* = 0.0411). 

#### 3.2.3. Lower Body 

**Figure 3:**
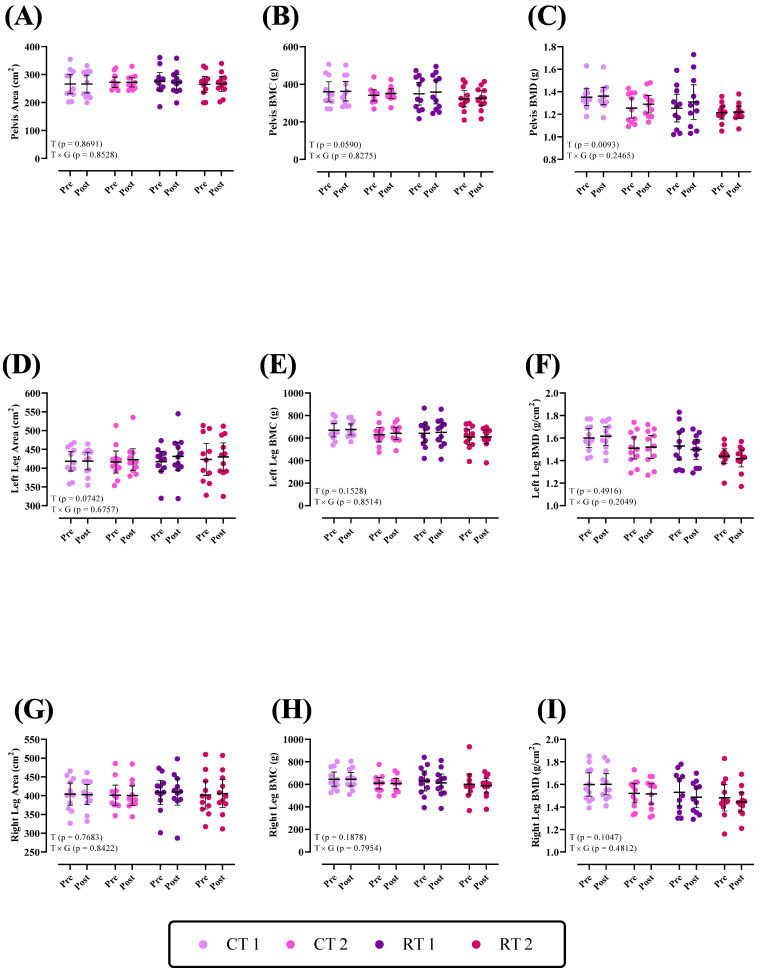
Effects of resistance or concurrent training in combination with high-protein diets on lower body bone parameters. *n* = 11 per group; error bars represent 95% confidence interval (CI). CT1, concurrent training + 1.6 g kg^−1^ d^−1^; CT2, concurrent training + 3.2 g kg^−1^ d^−1^; RT1, resistance training + 1.6 g kg^−1^ d^−1^; RT2, resistance training + 3.2 g kg^−1^ d^−1^; T, time effect; T × G, time × group interaction; BMC, bone mineral content; BMD, bone mineral density.

Changes in bone parameters of the lower body throughout the intervention are shown in Figure 3. There was no group × time interaction (*p* > 0.05) for the pelvis, left and right leg for the area, BMC, and BMD (*p* values are shown in each figure). There was a significant time effect for Pelvis BMD (*p* = 0.0093). 

## 4. Discussion

Our findings add to the existing body of research by demonstrating that high-protein diets (1.6 or 3.2 g kg^−1^ d^−1^), when consumed over 16 weeks combined with RT or CT, do not negatively affect whole-body bone parameters in resistance-trained males. This supports the notion that high-protein diets can be safely incorporated into the dietary regimens of athletes without compromising bone health. These results contribute to the understanding of the complex interplay between dietary protein intake, exercise, and bone health, emphasizing the importance of tailored nutritional and training strategies for optimizing bone health in this specific population. 

RT may have different effects on different types of bone tissue and different sites of the skeleton, depending on the magnitude, direction, and frequency of the strain applied [24,44]. Regarding the effectiveness of RT, previous studies reported different results depending on different variables. According to the National Strength and Conditioning Association (NSCA), intervention studies are less clear as to whether RT programs result in increases in bone mass [44]. There were no observed differences in Bone (re)modeling markers (BMM) between control participants and female athletes engaged in high-impact sports [45], rhythmic gymnasts [46], and male master runners and speed/power athletes [47]. However, these studies indicated that bone microarchitecture, also known as BMD, was affected by exercise. This implies that changes in bone mass or microarchitecture may not always be accurately reflected by BMMs [48]. Other studies indicated that non-impact sports, such as swimming, do not show an effect on improving BMD [49,50]. However, studies that incorporated swimming to reverse the pattern of bone loss caused by the lack of mechanical stimuli showed efficacy in increasing BMD after swimming [23,49,51]. Remarkably, previous research has shown that a 12-week RT program did not improve bone formation or prevent bone breakdown in young adult women. Similarly, maintaining a high-protein diet for 10 days in these women had no impact on bone metabolism [52]. While the results of these studies were consistent with our results, the meta-analysis and systematic review by Ponzano et al. (2021) showed that progressive RT alone or in combination with other interventions may improve BMD in individuals at risk of fracture [53]. However, progressive RT interventions or exercise adaptations appear to be more substantial and effective for older or less physically fit adults [54,55,56]. For example, a trial on healthy women with an average age of ~55 years who performed progressive, resistive back-strengthening exercises showed benefits on BMD after 10 years of follow-up compared to the control group [57]. Another study using the same high-intensity exercise intervention and RT in physically inactive, healthy young adult women with below-average bone mass reported similar findings [14]. Thus, participant age appears to be a significant factor in the capacity of exercise to induce any positive effects on bone mass. 

Several different possible pathways related to the effectiveness of RT on bone can be investigated. Bone tissue is significantly affected by strain, which is the deformation (bending) of bone caused by mechanical forces such as muscular contractions [58]. When bone tissue is strained, it rapidly stimulates bone cells to begin bone modeling, which involves the production of protein molecules that are deposited in the spaces between bone cells (59). These protein molecules increase the strength and density of the bone tissue, making it more resistant to fracture and osteoporosis [59]. Exercise influences bone responses through various metabolic signals, including reactive oxygen/nitrogen species [60], pH alterations [61], and the availability of serum calcium [62]. While some modeling may occur, it appears that remodeling is the primary mechanism through which the bone responds to exercise-induced mechanical or metabolic stimuli [63,64]. Another factor is the connection between bone and muscle [65,66,67]. Muscle tissue can affect bone health through the action of factors derived from muscle [13,68,69,70]. In particular, myostatin can regulate the activity of osteoclasts and bone destruction, and exercise can inhibit it [69]. In contrast, many other myokines, such as transforming growth factor-β, follistatin, insulin-like growth factor-I, fibroblast growth factor-2, osteoglycin, FAM5C, irisin, interleukin-6, leukemia inhibitory factor, IL-7, IL-15, monocyte chemoattractant protein-1, ciliary neurotrophic factor, osteonectin, and matrix metalloproteinase 2, that are regulated by exercise, can affect bone metabolism [69,70]. Myokines play a number of physiological functions, such as regulation of glucose metabolism, vascularization, and bone metabolism [71]. Muscle, being the largest organ in the body, releases myokines into the bloodstream that regulate the endocrine system of distant organs like bone [69].

As mentioned earlier, exercise plays a crucial role in maintaining BMC and BMD. Nevertheless, it is important to consider that there is little understanding of the impact of a concurrent regimen of chronic exercise training and a high-protein diet on several aspects related to bone health. Our current work demonstrated that a high-protein diet (3.2 g kg^−1^ d^−1^) had no adverse effects on bone minerals in resistance-trained men. Our findings align with recent studies that dismiss the notion of proteins’ negative effect on bone mass [26,72]. However, our results seem to contradict other research highlighting the positive influence of exercise on bone mass [73,74]. Similar to our results, Antonio et al. (2018) conducted a 6-month investigation that showed a high-protein diet (>2.2 g kg^−1^ d^−1^) had no adverse effects on BMD in exercise-trained women [75]. Studies that examine the association between protein intake and bone health parameters are limited and equivocal. For instance, there is evidence that protein helps keep bones healthy, as evidenced by Rizzoli et al. (2018), Dolan and Sale (2019), Langsetmo et al. (2018), and Darling et al. (2019) [27,76,77,78]. The effects of protein supplements along with exercise on bone health in different groups of people, such as apparently healthy men and women [79], healthy young adults [80], overweight and obese premenopausal women [81], and women after menopause [82], have been evaluated. These studies reported no change [80], a possible increase in both bone formation and breakdown [79], or only increased bone formation [81,82]. Nevertheless, the previous two trials administered other nutrients with protein, such as calcium [81] or CHO/calcium/vitamin D [82], making it impossible to isolate the specific impact of protein alone. A meta-analysis by Darling et al. (2009) did not find any effect of protein intake on the risk of breaking bones [28]; however, a more recent meta-analysis by Wu et al. (2015) showed a small decrease in hip fractures [29]. Early evidence indicated that a high-protein diet could potentially diminish BMD [83]. Also, research shows that a sufficient intake of protein is essential for the synthesis and maintenance of bone tissue, as well as for triggering the action of insulin-like growth factor 1, which enhances calcium assimilation and promotes bone growth. Nevertheless, the metabolic process of dietary sulfur amino acids, predominantly derived from animal protein, can result in heightened physiological acidity. This may have long-term adverse effects on bone health. Cereal foods, likewise, comprise dietary phytate, which is composed of phosphate. Phosphate consumption has been identified as a potential contributor to elevated physiological acidity. Consequently, cereal products may generate an equivalent amount of acid as animal proteins containing sulfur amino acids [84]. However, evolving research has provided a counter perspective, suggesting the beneficial effects of protein on bone tissue [85,86]. In the current investigation, despite the fact that the participants were assigned to two high-protein diets, we did not find any adverse impacts on bone tissue. Even in the group consuming 3.2 g kg^−1^ d^−1^ of protein, there was no observed decrease in BMD, although it is important to consider the effect of exercise along with two high-protein diets. The molecular mechanism(s) underpinning the effects of protein intake on bone health are complex and not yet fully elucidated. Consuming a substantial amount of protein has several beneficial benefits for both the skeletal and muscular systems. These effects include the provision of essential components for the development and maintenance of bone structure, as well as the augmentation of IGF-1 levels, which facilitates bone formation [87,88], lowering parathyroid hormone, which makes bones lose calcium and helps the body absorb more calcium from food [87,89], and keeping and improving muscle mass and strength [80]. Research conducted during the last decade has consistently shown that the temporary increase in protein consumption does not have detrimental effects on calcium equilibrium and skeletal well-being [80,87] and that consuming more protein for a long time is linked to higher bone density [90,91,92]. Nevertheless, there is a lack of consensus among many studies about this matter [93], maybe due to other dietary components [94]. However, it seems that the positive impacts of consuming a high-protein diet tend to counterbalance or surpass the negative impacts of increased urinary calcium excretion on bone health [6].

Ultimately, it is essential to acknowledge that these rationales are conjectural, and it is imperative to ascertain the specific elements that are responsible for the absence of discernible alterations in bone tissue. Participants’ age, sex, fitness level, and baseline bone mass can all influence the response to exercise interventions. Studies involving different populations, such as postmenopausal women, older adults, or athletes, may yield diverse results due to variations in hormonal profiles, bone turnover rates, and initial bone parameters. Also, differences in exercise protocols, such as the choice of RT exercises, load progression, rest intervals, or the inclusion of weight-bearing activities, may contribute to conflicting findings along with intervention duration. In this regard, bone remodeling is a slow process, and shorter intervention periods may not be sufficient to detect significant changes in bone density. Measurement techniques should also be considered. Variations in the methods used to assess bone mass, such as DXA, peripheral quantitative computed tomography (pQCT), or quantitative ultrasound, can contribute to discrepancies. Different measurement sites (e.g., spine, hip, and forearm) may also yield varying results. Despite rigorous study design and adherence to the established training and diet protocols, the anticipated correlation between the applied diets or training types and changes in bone parameters was not observed. These findings indicate that, within the conditions of this study, neither the high-protein diets nor the type of training significantly influenced bone parameters in our sample of resistance-trained males. It is important to acknowledge that the absence of substantial findings does not necessarily indicate the absence of a correlation between diet, exercise type, and bone health in resistance-trained men. Instead, it suggests that the specific parameters of our study may have been inadequate for uncovering such an impact. Our findings should be interpreted in the context of the existing literature, which has demonstrated varying effects of diet and exercise on bone health in different populations and under different circumstances. Future research should consider increasing the length of intervention or using more sensitive measures of bone health (such as plasma bone turnover biomarkers) to further explore this topic. 

## 5. Conclusions

Despite the absence of significant findings, our study is deemed to provide a valuable contribution to the existing body of research in this field. This is achieved via the inclusion of an additional data point and the provision of insights into methodological issues that might guide future studies. Ultimately, the investigation into comprehending the intricate interplay of diet, physical activity, and skeletal/bone well-being persists as an ongoing and formidable area of scientific investigation. Our work emphasizes the need for more exploration and urges the scientific community to persist in studying these associations using rigorous and creative methodologies. 

## Figures and Tables

**Table 1:** Baseline characteristics of the participants.

	CT1	CT2	RT1	RT2
Measure				
Anthropometry and training experience				
Age (year)	27 ± 6	25 ± 7	26 ± 6	28 ± 5
Height (cm)	178 ± 5	179 ± 8	180 ± 7	182 ± 6
Body mass (kg)	83.8 ± 10.6	81.6 ± 10.7	82.1 ± 9.1	85.2 ± 10.9
BMI (kg.m^−2^)	26.3 ± 3.4	25.2 ± 3.1	25.1 ± 2.3	25.7 ± 2.9
Training experience (year)	3.7 ± 2.2	4.6 ± 2.6	3.5 ± 1.7	4.8 ± 2.4
Bone parameters				
Left Arm Area (cm^2^)	231.5 ± 18.7	238.2 ± 36.6	233.6 ± 25.8	239.9 ± 36.5
Left Arm BMC (g)	228.1 ± 43.6	218.3 ± 42.3	213.9 ± 32.7	225.2 ± 50.7
Left Arm BMD (g/cm^2^)	0.98 ± 0.12	0.91 ± 0.08	0.91 ± 0.06	0.93 ± 0.08
Right Arm Area (cm^2^)	243.2 ± 17.03	243.4 ± 31.8	240.1 ± 24.1	247.7 ± 36
Right Arm BMC (g)	226.4 ± 23.4	220.5 ± 37.7	216.18 ± 34	228.3 ± 50.2
Right Arm BMD (g/cm^2^)	0.93 ± 0.06	0.90 ± 0.07	0.89 ± 0.08	0.91 ± 0.08
Left Ribs Area (cm^2^)	133.9 ± 8.7	138.7 ± 18.2	137.5 ± 17.4	138.5 ± 27.1
Left Ribs BMC (g)	116.6 ± 13.2	115.2 ± 25.6	113.6 ± 21.7	114.7 ± 29.2
Left Ribs BMD (g/cm^2^)	0.87 ± 0.07	0.82 ± 0.10	0.82 ± 0.08	0.82 ± 0.09
Right Ribs Area (cm^2^)	128.8 ± 17.8	138.3 ± 18.4	131.8 ± 18.4	137.5 ± 20.7
Right Ribs BMC (g)	114.8 ± 14.5	114.6 ± 18.9	111.6 ± 24	116 ± 20
Right Ribs BMD (g/cm^2^)	0.89 ± 0.06	0.82 ± 0.07	0.84 ± 0.09	0.84 ± 0.08
Thoracic Spine Area (cm^2^)	146.1 ± 11.2	144.1 ± 14.2	141.5 ± 11.3	156.8 ± 19.2
Thoracic Spine BMC (g)	163.6 ± 21.1	147.9 ± 20.4	148.7 ± 29.5	160.2 ± 46.5
Thoracic Spine BMD (g/cm^2^)	1.11 ± 0.10	1.02 ± 0.10	1.04 ± 0.14	1.01 ± 0.20
Lumbar Spine Area (cm^2^)	64.1 ± 3.9	64.6 ± 7.6	66.6 ± 6	68.3 ± 8.7
Lumbar Spine BMC (g)	76.9 ± 11.1	73.2 ± 12.4	76.4 ± 19.4	76.6 ± 21.6
Lumbar Spine BMD (g/cm^2^)	1.19 ± 0.12	1.13 ± 0.13	1.13 ± 0.21	1.10 ± 0.20
Pelvis Area (cm^2^)	265.9 ± 51.1	272.4 ± 27.7	275.8 ± 46.9	264.9 ± 42.7
Pelvis BMC (g)	360.1 ± 79.6	341.2 ± 44.2	349.1 ± 89.5	322.7 ± 65.1
Pelvis BMD (g/cm^2^)	1.35 ± 0.11	1.25 ± 0.13	1.25 ± 0.18	1.21 ± 0.08
Left Leg Area (cm^2^)	418.3 ± 38.6	416.3 ± 43.7	417.7 ± 39.7	423.2 ± 63.1
Left Leg BMC (g)	669.9 ± 89.2	629.7 ± 96.3	642.5 ± 122.3	610 ± 102.5
Left Leg BMD (g/cm^2^)	1.59 ± 0.12	1.51 ± 0.14	1.52 ± 0.18	1.43 ± 10
Right Leg Area (cm^2^)	404.2 ± 43.9	401.3 ± 39.6	408.6 ± 47.7	401.4 ± 54.9
Right Leg BMC (g)	646.1 ± 93.8	610.1 ± 75.8	630.1 ± 132.1	599.1 ± 137
Right Leg BMD (g/cm^2^)	1.59 ± 0.15	1.52 ± 0.12	1.52 ± 0.17	1.48 ± 0.17

Values are presented as mean ± standard deviation. Abbreviations: BMC, bone mineral content; BMD, bone mineral density; CT1, concurrent training + 1.6 g kg^−1^ d^−1^; CT2, concurrent training + 3.2 g kg^−1^ d^−1^; RT1, resistance training + 1.6 g kg^−1^ d^−1^; RT2, resistance training + 3.2 g kg^−1^ d^−1^.

## Data Availability

The data that support the findings of this study are available on request from the corresponding author.
